# Myths and misconception of COVID-19 among hospital sanitary workers in Pakistan: Efficacy of a training program intervention

**DOI:** 10.1186/s12913-022-08217-6

**Published:** 2022-06-24

**Authors:** Jamil Ahmad Malik, Sadia Musharraf, Razia Safdar, Mazhar Iqbal

**Affiliations:** 1 National Institute of Psychology, Quaid-i-Azam University, Islamabad, 45320 Pakistan; 2 Department of Applied Psychology, Women University, Multan, Multan, Punjab Pakistan; 3 Ministry of National Health Services, Regulations & Coordination, Islamabad, Pakistan; 4 Department of Environmental Sciences, Quaid-i-Azam University, Faculty of Biological Sciences, Islamabad, 45320 Pakistan

**Keywords:** COVID-19, Sanitary staff, Myths, Training intervention, Frontline fighters

## Abstract

**Objective:**

Hospital sanitary workers are among the prime source to disseminate information at a massive level, however they received least attention during the pandemic COVID-19. The study was designed to investigate the prevailing myths and misconceptions of the coronavirus pandemic among the sanitary workers of health care system. Further, a systematic training program is devised and tested to demystify the false myths with discerning truth and awareness-raising in hospital sanitary workers.

**Method:**

A pre-post face-to-face intervention design was opted and the intervention was conducted at five locations by the project team. The intervention consisted a 3 days training program to target myths and misconceptions of hospital sanitary workers. The study was completed in 8 months starting from August, 2019 to March, 2020. Participants were recruited from local hospitals having a specialized indoor COVID treatment facility. The sample consisted of 82 participants (*n* = 25, 30.09% females) with age ranging from 18 to 60 years (M ± SD = 37.41 ± 10.09).

**Findings:**

The results indicated that 86.4% of the participants never heard the name of the coronavirus before the pandemic in Pakistan. A majority of the participants (> 50%) believed on a very alarming but unrealistic rate of mortality i.e., 30–60%. The pre-testing showed a high prevalence of myths in all four domains (i.e., popular treatments = 24.44, conspiracy myths = 7.93, home remedies = 16.46, and COVID-reliance = 7.82). The pre and post comparison of individual myths showed significant improvement on 24 of the 26 myths with a decline ranging from 0.18 to 1.63. Overall, the intervention significantly decreased scores on all four domains of coronavirus myths.

**Conclusion:**

The training intervention appeared to effectively reduce myths and misconceptions of sanitary staff workers and is advised to be included as a standard training program for sanitary workers of health care system.

## Introduction

Myths are traditional stories or narratives that are generally based on fictional origins without any scientific evidence. However, they serve a fundamental role in shaping beliefs, attitudes, and behaviors of people in a society. Recently, a wide range of myths and incorrect assumptions emerged with the explosive spread of the COVID-19 pandemic. A large amount of scientific literature [1–3] and guidance about the novel COVID-19 is available on the web pages of various health organizations [4, 5]. However, COVID-19 associated false myths and misinformation are equally fast-spreading like the COVID-19 infection itself.

Serious concerns have been raised about the risks of viral transmission of Coronavirus from infected individuals to healthcare workers as they are in close contact with infected cases at their workplaces [6, 7]. Likewise, evidence indicated that the COVID-associated mortality and infection rate of healthcare workers is rapidly rising across the world due to their occupational exposure [6, 8]. Health care workers include doctors, nurses, technicians, paramedics, and non-clinical staff such as patient navigators, security personnel, housekeepers, attendants, and sanitary workers. Although, an influx of evidence-based literature on the risks and safety of health care workers is available, however, most studies only focused on the medical staff, more particularly physicians and nurses [9–11]. Until now, little attention has been given to the safety of sanitary workers that are equally vulnerable to contract the virus as they are responsible for infectious waste management [12]. A study conducted by Swapnajeet, et al., found that a substantial portion of sanitary workers reported several negative emotional states including anxiety and stress [13].

Sanitary workers in developing countries belong to a low socio-economic group and have low levels of education [12]. Research has demonstrated that the low level of education, knowledge, and non-inclusive approach towards training has a significant influence on the dissemination of rumors [14]. Empirical evidence indicates that interpersonal communication leads to higher perceived trustworthiness of false myths and a greater willingness to spread them in comparison to mass communication [15]. Moreover, when false myths are promulgated by the people working in the health care settings, it has a strong influence on the community. Additionally, debunking myths and misconceptions of people with low education yet having direct field experience is always a challenge. These challenges can only be met by developing and delivering intensive training programs tailored for people with low educational profile and rigid approach.

Research revealed that health care workers can stop the spread of false myths by debunking misinformation and by providing accurate information about the pandemic [16, 17]. Therefore, training the non-medical health care workers to learn the facts can assist in controlling the spread of fake information and false myths in the community. Therefore, the present research is conducted to test an intervention plan addressing COVID-19 related myths and misconceptions of hospital sanitary workers. Although, there is a substantial amount of literature on the myths/misinformation associated with COVID-19 and its potential impacts [18], no research has been conducted to date that evaluates the efficacy of systematic training program to demystifying the false myths with discerning truth and awareness-raising in hospital sanitary workers.

This study was designed for hospital sanitary workers of Pakistan. The country has a population of 221 million with 62% literacy rate and a majority i.e., 96.5% following Islam as religion. At the time of the study, vaccine was not available and no vaccination drive was yet started. Pakistan is a low-middle income country with a very low budget (3.5% GDP) for health. COVID-19 facilities were developed on a priority basis in the public sector hospitals as the cost of care was very high in private hospitals ranging from US $ 2000 to $ 10,000. This cost is unaffordable to average people as there was no national health insurance system at the time of pandemic. A full lockdown was initiated for first 3 months of the pandemic and that was gradually converted into a smart/partial lockdown. When the study was initiated, there was a scarcity of PPEs however in the final stages of intervention most participants reported complete access and provision of PPEs by their respective hospital administrations. The participants worked in any of the three shifts (i.e., morning, evening, and night) with approximately 10% working for 5 days a week, 55% working for 6 days a week and 35% working for all 7 days a week. Their responsibilities included waste collection from wards to disposal at the collection points. The participants were not in a direct contact to patients; however they cleaned COVID-19 facilities including patients’ utilities.

Myths and infodemic may contribute to mitigating fear and undermining public trust in health agencies and governments [19]. For example, a bizarre myth was circulating on social media that medical professionals and local governments are being paid by WHO for falsely declaring patients as COVID-19 infected [20]. In response to this misinformation, many sick patients delayed consultations and approached hospital with advanced Coronavirus disease. Thus, a large number of patients died either in ambulances or shortly after arrival in the hospital [21]. Further, inundation of misconceptions concerning cure and prevention of COVID-19 disease may increase risk behaviors which consequently intensify the actual crisis. For example, in Iran, 800 people died because of drinking industrial-grade alcohol after the circulation of a false claim that alcohol intake could cure or protect one from COVID-19 [22]. Similarly, in Qatar, two men ingested hand sanitizer following misinformation that it could aid in protection against COVID-19 associated infection [23]. In India, 12 villagers took poisonous seeds named “Datura” after watching a video on social media suggesting that these seeds boost immunity to fight against Coronavirus infection [24].

There is no empirical evidence indicating that such supplements can prevent COVID-19 [25]. Moreover, there was a big leap of faith over a large number of home remedies for the elimination of this new virus. These include steam inhalation, gargling with warm water, salt solution, and vinegar [26], consumption of herb leaves “sana makki,” which has laxative properties [27, 28], eating ginger, garlic, and black seeds [20]. Besides nutritional benefits, self-medicating with food supplements, herbal concoctions and various spices is unsafe and may cause serious health risks due to incorrect dosage, naturally occurring toxins in herbs, allergic reactions, and unknown drug interactions [25, 29]. When myths and false claims tagged with influential names circulate in populations having low health literacy, individuals may poison themselves in an effort to self-medicate [25]. This study is designed with the assumptions that busting myths of health care workers may have a huge impact on the myths of general population. More specifically, the study has following aims. First, to determine and estimate prevalent myths and misposition of COVID-19 among hospital sanitary workers. Second aim is to develop and test the efficacy of a training program Intervention for myths and misposition of COVID-19.

## Method

### Design

The present study used a pre-post intervention design. The study was approved by the Bioethic Committee of the Faculty of Biological Sciences, Quaid-i-Azam University, Islamabad with ethical approval reference no. BEC-FBS-QAU ONL-2020- 001. The study was completed in 8 months starting from August, 2019 to March, 2020.

### Sample

The sample was recruited from hospitals of five cities (Four Provincial Capitals and 1 Federal Capital). The inclusion criteria were that the hospital shall have COVID-19 ward/quarantine facility, and the participants shall be a permanent employee performing his/her duties as a sanitary worker and involved in COVID-19 related waste management, inside or outside the COVID-19 ward. Participants suffering from or taking treatment for a medical or psychological condition were not included in the study. Informed consent was obtained from all individual participants included in the study. The final sample consisted of 82 participants (*n* = 25, 30.09% females) with age ranging from 18 to 60 years (M ± SD = 37.41 ± 10.09). Majority of the participants (*n* = 67, 82.7%) were married. A total of 16 participants were illiterate and the same number of participants has a primary level of education. Maximum i.e., *n* = 21 participants were under-matric, and 14 participants completed their matriculation. Twelve participants have a college degree, and one participant has a master’s degree. The monthly income of the participants ranged from 10 to 45 thousand (PKR) with M ± SD = 24.40 ± 8.12.

### Procedure

**Fig. 1:**
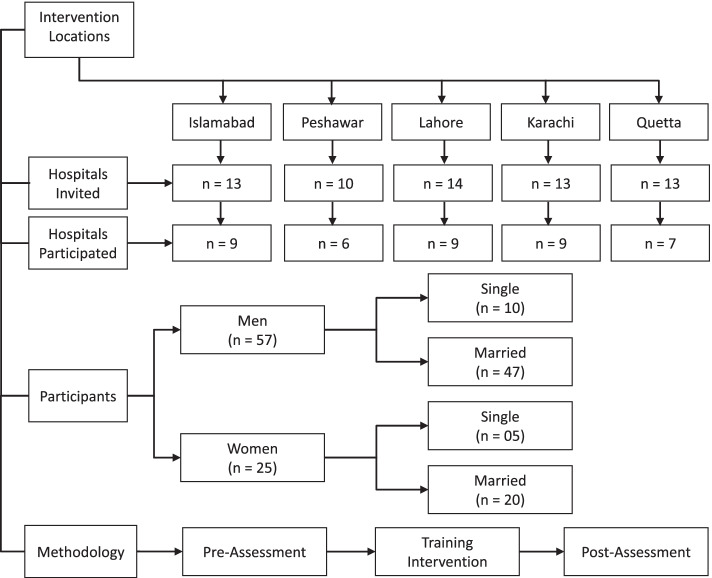
Explaining recruitment of participants and methodology

All hospitals that met the criteria in all five cities were invited to nominate two participants (1 male and 1 female, wherever possible) wide an invitation letter containing the information about training sessions through the Federal Health Ministry followed by telephonic confirmation by the research assistant at Quaid-i-Azam University. It would have been more desirable to accommodate all eligible participants, however due to financial restraints of this pilot project, hospitals were instructed to nominate only two participants. Each of the 40 participating hospitals (9 Islamabad/Rawalpindi, 6 Peshawar, 9 Lahore, 9 Karachi, and 7 Quetta) nominated two participants (few nominated 3) for the said training (detail elaborated in Fig. 1). A day before the trainings, nominated participants were individually called on their cell phone numbers to give them a brief introduction about the training to seek their informed consent. The PI (Principal Investigator), Co-PI (Co- Principal Investigator), three qualified trainers, and 2–3 research scholars (depending on the group size of participants) were engaged from local universities to help in assisting pre and post-testing for the illiterate participants. The PI and the Co-PI were responsible to observe the training sessions and maintain same standards across training stations. The health safety of the trainers, team members/data collectors, and participants were ensured by following the standard operating procedures (SOPs).

### Intervention

The training consisted of 2 days, three sessions each day. The program was reviewed by the PI and the Co-PIs, followed by independent discussion sessions with each trainer. The modifications suggested by the PI and the Co-PIs were incorporated in the training program by the trainers. The finalized training program included six modules. Day one consisted of three sessions with [1] Information about COVID-19, [2] Safety Practices, and [3] Psycho-social effects. The three sessions of the second day included [1] Myths and misconceptions, [2] Waste management, and [3] Dealing with stressors. The trainers used interactive sessions including practice of hands-on skills for the participants. The sessions were conducted same hall, and included lectures with audio-visual materials, discussions, individual and group activities considering the low levels of participants’ education.

### Instruments

In the first stage, a thorough review was conducted to develop a comprehensive questionnaire. A team of experts consisting of the PI, the Co-PIs, and three trainers conducted several brainstorming sessions to develop various segments of the questionnaire. The team reviewed the research literature published during the pandemic using the keywords, Corona, and COVID-19. Further, the experts reviewed various guidelines and policy documents of both national and international programs. The final questionnaire consisted of three sections (a) basic knowledge, (b) safety measures (c) myths and misconceptions, along with a detailed demographic sheet and informed consent form. Parallel versions of the questionnaire were developed in both English and Urdu language, however only Urdu version was used in both pre- and post-assessments.

### COVID-19 related myths and misconceptions (my-COV)

The rating scale to measure COVID-related myths and misconceptions was developed by the researchers and validated on an independent sample of university students. The scale was developed by the PI and the Co-PIs and reviewed by the trainers for content relevance and comprehension. The suggestions were incorporated and pre-testing was conducted on five sanitary workers. The scale consisted of 27 items with a 4-point Likert-type response option wherein “1= absolutely wrong” and 4 = absolutely correct”. After resolving the difficulty level and comprehension issues, the scale was validated on a sample of 225 university students in an independent study. Final scale consisted of 24 items and measured COVID-19 related myths across 4 domains labeled as [1] Popular Treatments (11-items, α range = .64–.65), [2] Home Remedies (6-items, α range = .51–.77), [3] COVID-19 Resilience (4-items, α range = .51–.56), and [4] Conspiracy Myths (4-items, α = .59–.71).

## Results

The preliminary analysis showed that 86.4% of the participants never heard the name of Coronavirus before the outburst of the pandemic in Pakistan. More than 60% of the participants answered “no” for the question stating “were there any pandemics in the world similar to corona”? However, only 19 of the 31 answering “yes” wrote names i.e., Dengue virus, (*n* = 5), Congo virus, Swine flu, and Pneumonia (*n* = 2 each). Among the pandemic names given by only one of the participants included Ebola virus, Polio, Asthma, Influenza, Mata, Pimples, Plague, and Typhoid.

Regarding spread of corona, 40% of the participants rated less than or equal to half of the world’s population, 26% reported that 60–90% of the world’s population will be affected while the reaming 24% of participants said that 100% population of the world will be affected by coronavirus. Further, 32% participants reported that less than 30% of the affected population will die, 40% of the participants indicated a death rate of more than 30% but less than 50%, and the remaining 28% reported a very high impact with more than 60% death rate. A small number of participants had the misconception that coronavirus is named after the area from where it emerged and a large group approximately 72% participants thought that corona is named due to its effect. Only (*n* = 16) approximately 20% of the participants knew that coronavirus is named due to its structure. Further, the majority of the participants about 57% had the misconception that all viruses affect humans, and 62% thought that all viruses are dangerous.

To test the effect of training intervention, a comparison of myths was conducted by paired sample t-test between pre and post measurement. The results presented in Table 1, show a significant decline in the perception of myths due to training intervention among the participants. The mean score significantly decreased on all four dimensions of myths with approximately 10 points decline on popular treatments, 3 points decline on conspiracy myths, 5 points decline on home remedies related myths, and two points decrease on COVID-19 resilience myths. The pre- and post-comparison of individual myths showed significant improvement on all eleven popular treatment related myths, with a decline ranging from 0.18 to 1.63. The highest mean difference (MD = 1.63) is related to warm baths followed by fly and mosquito as a carrier (MD = 1.56), and sunlight (MD = 1.25) as effective treatments.

**Table 1:** Paired sample t-test to compare score on myths between pre and post assessments

Variables/Indicators	Pre		Post		*t*	*p*	95% *CI*		*r*	*p*
	*M*	*SD*	*M*	*SD*			*LL*	*UL*		
Popular Treatments	24.44	6.88	14.99	4.44	12.65	.00	7.97	10.94	0.38	0.00
Alcohol	1.65	1.12	1.14	0.60	4.10	.00	0.26	0.75	0.33	0.00
Antibiotic	2.59	1.29	1.63	1.03	5.75	.00	0.63	1.29	0.23	0.05
Sunlight	2.97	0.97	1.72	1.01	9.72	.00	0.99	1.51	0.36	0.00
Diluted Bleach (drinking)	1.23	0.66	1.05	0.32	2.21	.03	0.02	0.34	0.07	0.56
Hot Air	1.71	1.04	1.17	0.66	4.42	.00	0.30	0.79	0.25	0.03
Breath Holding (10 Sec)	2.27	1.22	1.34	0.84	6.93	.00	0.67	1.20	0.39	0.00
Warm Bath	3.29	4.85	1.66	1.00	2.99	.00	0.55	2.72	0.20	0.09
Spicy Soups	1.78	1.04	1.39	0.83	3.55	.00	0.17	0.60	0.52	0.00
Fly & Mosquito as Carrier	2.75	1.22	1.19	0.69	10.50	.00	1.26	1.85	0.15	0.19
Side-effect of Mask	2.46	1.14	1.66	1.01	5.03	.00	0.48	1.11	0.20	0.09
Sitting in front of Fire	1.74	0.96	1.10	0.41	5.49	.00	0.41	0.87	0.03	0.76
Conspiracy Myths	7.93	2.99	5.65	2.27	7.42	.00	1.67	2.89	0.50	0.00
5-G Technology	2.33	1.20	1.42	0.91	5.35	.00	0.57	1.25	0.04	0.72
Business of Fear	1.91	1.18	1.52	0.98	3.07	.00	0.14	0.64	0.48	0.00
Controlling Fertility	1.76	1.06	1.24	0.65	4.31	.00	0.28	0.77	0.30	0.01
Hidden Treatment	1.94	1.17	1.48	1.03	3.19	.00	0.17	0.74	0.36	0.00
Home Remedies	16.46	3.71	11.45	3.96	9.71	.00	3.98	6.04	0.31	0.01
Bleach (on floor/surface)^a^	2.91	1.19	2.17	1.25	3.89	.00	0.36	1.11	0.08	0.48
Steam	3.45	0.79	2.62	1.08	6.06	.00	0.56	1.10	0.22	0.06
Chloroquine	2.59	1.00	1.63	0.99	6.71	.00	0.68	1.25	0.21	0.07
Garlic	2.38	1.08	1.73	1.00	4.75	.00	0.38	0.93	0.32	0.00
Ginger with Honey	2.63	0.95	1.70	1.02	7.41	.00	0.68	1.19	0.38	0.00
Sana Makki Tea	2.42	1.10	1.61	0.95	6.04	.00	0.54	1.07	0.36	0.00
COVID-19 Resilience	7.82	2.89	5.85	2.03	5.93	.00	1.31	2.63	0.33	0.00
Only Children	1.30	0.78	1.26	0.75	0.37	.71	−0.17	0.25	0.27	0.02
Only Old People	2.06	1.24	1.55	0.95	3.23	.00	0.20	0.83	0.20	0.08
Pneumonia Vaccination	2.00	1.14	1.18	0.62	5.54	.00	0.52	1.11	0.00	1.00
Multi-Vitamin	2.45	1.14	1.83	0.99	4.35	.00	0.33	0.90	0.31	0.01
Cold Area	1.32	0.84	1.15	0.60	1.59	.12	−0.04	0.38	0.27	0.02
Recovery Rate^a^	3.39	0.80	3.17	1.01	1.60	.11	−0.05	0.50	0.11	0.33

(^a^) indicates reverse coded item

Among the conspiracy myths, the highest decline is observed for the 5-G technology as the cause of the virus (MD = .91) followed by myth related to controlling fertility (MD = 0.53). A substantial decline is observed on all the myths related to home remedies with mean differences ranging from 0.65 to 0.96. The highest decline is observed on the myth related to chloroquine as an effective treatment. Among the COVID-19 resilience related myths, highest decline is observed in the beliefs for resistance against coronavirus due to pneumonia vaccination (MD = 0.82) followed by use of multi-vitamin (MD = 0.62). Finally, no significant decline is observed on the two ungrouped myths (i.e., *p* > .05) for myth related to cold places for treatment and myth related to high recovery rate.

## Discussion

It is expected that in health care system sanitary workers are very vulnerable to develop and propagate misinformation due to inaccessibility of customized training programs targeting this population. Sanitary workers directly observe patients, yet their observation is narrowed to find evidence that may support the unrealistic information reaching to them. Both the mitigated information reaching to this population accompanied by their narrowed observation makes them confident and influential advocates of myths and misconceptions among masses. Thus, targeting myths of sanitary workers in health care system is expected to have an exponential impact on the community in a developing economy. The present study was devised as a pilot project to develop and test an intervention plan to address COVID-19 related myths and misconceptions of sanitary workers. The training was conducted at five stations including provincials and federal capital. To maintain standardization of the training program, same project team including trainers organized all five training programs.

The results of the study supported our assumption regarding the limited sources of information for the sanitary staff workers as a large majority of the participants indicated that they didn’t hear the name of the coronavirus before the outburst of the pandemic in Pakistan. Due to their low educational background and limited access to information, sanitary staff workers of low-income countries are mostly unaware and unconcerned about most developments in universal health care systems. Additionally, the results also confirmed the self-interpreted understanding of participants about the coronavirus as they indicated similarity of coronavirus to that of Dengue, Congo, Ebola, Polio, Swine flu, Influenza, Pneumonia, Asthma, Mata, Pimples, Plague, and even Typhoid. Further, the participants believed a very high mortality rate of coronavirus. This might have been partially due to their narrowed observation in hospital settings with only critical patients. As the lack of public trust [19] due to the rumor of injecting lethal substances [30] for sake of international funding [20] caused avoidance on seeking medical advice and resulting in critical patients only reaching to hospitals [21].

The result further confirmed that most of the basic knowledge about the name, diagnosis, effect, and treatment of coronavirus is based on their own interpretation. The main goal of the training intervention was to target myths of sanitary staff workers. The results of pre-testing showed that warm baths and steam were the highest-rated remedies of coronavirus by the sanitary workers. These beliefs have been commonly reported by many others [31, 32]. However, results of the post-assessment showed a substantial decline. Additionally, the training intervention appeared to be very effective in correcting false belief of mosquitos as carriers, and exposure to sunlight [33] as a treatment of the virus. Though some studies indicated a correlation between exposure to sunlight and recovery rate [34] however more research is needed to clarify and establish sunlight and vitamin D as preventive measures of COVID-19 [34–36].

Literature suggests that effectiveness of antibiotics is related to bacterial co-infection for COVID-19 patients [37, 38]. Results of the post-testing showed a substantial decline in the use of antibiotics as treatment to coronavirus. Following the common beliefs, the sanitary staff also rated breath-holding as a valid screening for COVID-19 [39], however, the intervention program busted this myth very effectively among the participants. Side effects of long-term mask usage have been propagated on social media [40]. The training intervention effectively decreased myths related to the side effects of wearing masks. This is an important achievement of the intervention program as recent empirical literature is rather suggesting potential benefits of mask-wearing in everyday life [41].

Various models have been tested and adopted to take prevention and protection measures of the health care workers across the globe. Intervention studies conducted in Italy indicated that early detection of symptomatic health care workers and the correct use of PPE were very helpful in avoiding nosocomial clusters [42, 43]. It was also evidenced that strict measures for PPE use and mass surveillance appeared as successful strategies to prevent infection among health care workers [44]. The safety protocol for the health care workers adopted in another study in the Wuhan showed effective control of the nosocomial infection [45]. None of these studies, however addressed myths and misconceptions of sanitary staff working in hospital settings.

The outbreak of the pandemic accompanied a variety of conspiracy myths including 5-G technology, controlling humans using chips, and controlling fertility using vaccines [46]. The training intervention appeared to be effective in decreasing the influence of such conspiracy myths among the sanitary staff workers. The home remedies myths to treat coronavirus including use of garlic, ginger, honey, and herb tea [47, 48] were also effectively addressed by the intervention. Though studies showed the nutritional effects of these diets [49], yet no empirical data support the therapeutic impact of such home remedies [50].

### Limitations and recommendation

The study has valuable findings, however as the study was based on a pilot project that included only two participants from each hospital, the prevalent myths and misconception may not be representative of the all sanitary-staff workers of the health care system in Pakistan. Given that the efficacy of the training program intervention is supported from this pilot project. It is therefore recommended that this program shall be replicated on a larger sample to grasp a more representative picture. Further customized intervention shall also be designed and tested for other hospital staff i.e., housekeeping, security personals, and paramedic staff.

## Conclusions

It is evidenced that sanitary staff workers in health care system are very vulnerable to develop and propagate myths and misconceptions. Further, it is concluded that the comprehensive intervention plans tailored to effectively address myths and misconceptions of low educated frontline fighters of health care system are inevitable.

## Data Availability

All data and analysis output are available with the first and corresponding authors and will be shared on request. However the data is not publicly stored in accordance to the confidentiality statements on the informed consent form that was signed by the participants.
